# Identification of Bioactive Metabolites from Dust-like Seeds of *Cremastra appendiculata* via Metabolomics, UPLC-Q-TOF-MS/MS, and Molecular Networking

**DOI:** 10.3390/plants15152399

**Published:** 2026-08-05

**Authors:** Zhao Liu, Yuan Chen, Kai-Peng Liu, Ruo-Xuan Xu, Gang Ding, Yan-Duo Wang

**Affiliations:** 1 State Key Laboratory of Bioactive Substance and Function of Natural Medicines, Institute of Medicinal Plant Development, Chinese Academy of Medical Sciences and Peking Union Medical College, Beijing 100193, China; s2024009050@student.pumc.edu.cn (Z.L.); 17732790202@163.com (Y.C.); s2025009055@student.pumc.edu.cn (K.-P.L.); 13589963816@163.com (R.-X.X.); 2 Department of Pharmacy, The First Affiliated Hospital of Army Medical University, Chongqing 400038, China

**Keywords:** orchid seeds, *Cremastra appendiculata*, metabolomics, UPLC-Q-TOF-MS/MS, molecular networking, symbiotic fungi, *Coprinellus disseminatus*

## Abstract

Orchid seeds are dust-like and lack endosperm, which limits their capacity to support germination using endogenous nutrient reserves. Successful germination therefore depends on the establishment of a compatible symbiotic association with germination-promoting orchid mycorrhizal fungi (OMF). In plant-root symbioses, host-derived small molecules, such as strigolactones and flavonoids, function as early chemical signals that recruit microbial partners and stimulate their growth. However, the chemical constituents that may facilitate fungal recognition, growth, or colonization during orchid seed germination remain poorly understood. In this report, we combined metabolomics, UPLC-Q-TOF-MS/MS, molecular networking, phytochemical isolation, and bioactivity assays to characterize bioactive metabolites from the seeds of the medicinal orchid *Cremastra appendiculata*. Metabolomic profiling revealed abundant primary metabolites, including lipids, amino acids, organic acids, saccharides, and nucleosides. And then, nineteen secondary metabolites were isolated and identified, including two new structures. Functional assays showed that selected organic acids, saccharides, and lignanamides promoted the growth of *Coprinellus disseminatus*, a fungus required for seed germination, whereas lignanamides inhibited the plant pathogen *Fusarium oxysporum*. These findings provide the first systematic chemical and functional characterization of metabolites from *C. appendiculata* seeds and offer new insight into the molecular basis of symbiosis between orchids and fungi.

## 1. Introduction

Orchidaceae are characterized by specialized symbiotic interactions with fungi. Orchid seeds are minute, lightweight, and typically wind-dispersed; a single capsule can produce tens of thousands of seeds [1]. However, these seeds lack endosperm and contain only limited reserves of primary metabolites, such as sugars, fatty acids, and proteins, and they are unable to germinate independently under natural conditions. Successful germination therefore depends on nutritional support from orchid mycorrhizal fungi (OMF). Histological studies have shown that germination requires the colonization of seed tissues by fungal hyphae, which provide nutritional resources during the early developmental stages [2,3]. Accordingly, the early interaction between orchid seeds and compatible germination-promoting fungi is critical for the establishment of a mutualistic symbiosis [4,5].

In addition to the symbiotic association between orchids and their germination fungi, symbioses also exist in nature between plants and arbuscular mycorrhizal (AM) fungi, as well as between leguminous plants and rhizobia. During AM symbiosis, plant roots secrete strigolactones, a class of secondary metabolites that stimulate spore germination, hyphal branching, and hyphal elongation in mycorrhizal fungi. In response, AM fungi release mycorrhizal factors, including lipo-chitooligosaccharides (LCOs), which activate symbiosis-related genes in the host and promote the establishment of mutualistic associations [6]. Similarly, legume roots secrete flavonoids that act as chemical signals to recruit rhizobia. After sensing flavonoids, rhizobia release Nod factors, which are also LCO-type signaling molecules that are recognized by the host plant and induce root nodule formation [7]. Although strigolactones and flavonoids have been established as key host-derived signals in AM and rhizobial symbioses, the corresponding chemical signals used by orchids to recruit or stimulate their germination fungi remain largely unresolved. Previous studies have confirmed that OMF can supply host plants with nutrients. A wide range of metabolites have also been isolated from orchids and their symbiotic fungi; however, bioactive small molecules from orchid seeds that directly promote the growth or recruitment of germination-promoting fungi have rarely been characterized [8].

*Cremastra appendiculata* is a perennial medicinal orchid and a rare, endangered species listed as a National Class II Protected Plant in China. Its dried pseudobulbs are used as a traditional Chinese medicinal material [9]. Gao et al. [10] isolated the mycorrhizal fungus *Coprinellus disseminatus* from *C. appendiculata* and demonstrated that this strain significantly improved seed germination, providing an important basis for the artificial propagation and conservation of this species. Although numerous metabolites have been isolated from the pseudobulbs of *C. appendiculata* and from orchid-associated fungi, systematic studies on seed-derived metabolites that may mediate fungal colonization, fungal growth, or symbiotic germination remain limited.

In the present study, we integrated metabolomics, UPLC-Q-TOF-MS/MS-based molecular networking, and phytochemical isolation to characterize metabolites from *C. appendiculata* seeds. Primary metabolites, including amino acids, nucleosides, lipids, organic acids, and saccharides, were identified by metabolomic profiling, while secondary metabolites were annotated and prioritized through molecular networking. Bioactivity assays were conducted to explore the growth effects of key compounds on *Coprinellus disseminatus* and *Fusarium oxysporum*. These findings establish a chemical basis for further investigation of bioactive molecules involved in *Cremastra appendiculata*–*Coprinellus disseminatus* symbiosis.

## 2. Materials and Methods

### 2.1. General Experimental Procedures

IR and CD spectra were recorded using an FTIR-8400S spectrophotometer (Shimadzu, Kyoto, Japan) and a J-815 spectropolarimeter (JASCO, Tokyo, Japan), respectively. UV spectra and optical rotations were measured on a UV-2102 spectrophotometer (Unico, Shanghai, China) and a 241 polarimeter (PerkinElmer, Waltham, MA, USA), respectively. NMR spectra were acquired on a Bruker AVANCE III 500 MHz spectrometer (Bruker, Billerica, MA, USA). Chemical shifts are reported as δ values and referenced to residual CD_3_OD signals (*δ*_H_/*δ*_C_ 3.31/49.0) and DMSO-*d*_6_ (J&K Scientific Ltd., Beijing, China) signals (*δ*_H_/*δ*_C_ 2.50/39.5). HR-ESI-MS data were obtained using a Waters Xevo G2-S QTOF mass spectrometer (Waters, Milford, MA, USA). Semi-preparative HPLC was performed on a SEP LC-52 system (Separation (Beijing) Technology Co., Ltd., Beijing, China) equipped with an MWD UV detector and a YMC-Pack ODS-A column (5 μm, 250 × 10 mm; YMC, Kyoto, Japan).

### 2.2. Sample Preparation and Metabolomics Analysis

#### 2.2.1. Seed Extraction

Five thousand fruit capsules of *C. appendiculata* were obtained from Chishui Shouhu Dendrobium Planting Co., Ltd. (Guizhou, China). The plant materials were legally sourced. This endangered species is propagated through micropropagation, an efficient method for the mass-scale propagation of rare, endangered orchids, and also for the sustainable extraction of their phytochemicals.

All 5000 *C. appendiculata* capsules used in this experiment were fully mature and at the pre-dehiscence stage. Intact mature capsules were transferred to a laminar flow sterile clean bench (ZHJH-C1106B, Donglian Electronic Technology Development Co., Ltd., Harbin, China). Sterile scalpels were used to gently cut open the pericarp along the longitudinal sutures of the capsules. Sterile brushes were employed to lightly sweep the internal septa of the capsules, allowing all powdery seeds to fall off completely for collection into sterile sealed containers. Impurities such as pericarp fragments were removed, and only purified seeds were retained for subsequent experiments.

The mature seeds of *C. appendiculata* (total weight 90 g) were ultrasonically extracted three times with 75% EtOH (Beijing Chemical Reagent Company, Beijing, China) at a controlled temperature of 25 °C, with the ethanol extract renewed every 6 h. All extraction solutions were combined and concentrated under reduced pressure using an EYELA N-1300 rotary evaporator at a constant water bath temperature of 30 °C under a vacuum of −0.08 to −0.09 MPa until the solvent was completely removed to yield the total crude extract (33.5 g). The total extract was thoroughly suspended in 300 mL of ultrapure water, and the aqueous suspension was successively partitioned with ethyl acetate (300 mL, Beijing Chemical Reagent Company, Beijing, China) and n-butanol (300 mL, Beijing Chemical Reagent Company, Beijing, China). The corresponding organic phases were concentrated separately to afford the EtOAc extract (17.0 g) and *n*-BuOH extract (8.0 g), respectively.

Initially, three parallel extracts were prepared from *C. appendiculata* seeds with the same extraction protocol. The overall HPLC chromatographic profiles and the distribution of major characteristic absorption peaks of each group were compared, and Pearson correlation analysis was performed. No obvious shifts in compound composition were observed among the three extracts. These results verified favorable repeatability between extracts (Figure S1). Considering the scarce resources of *C. appendiculata* seeds and the limited seed output from artificial cultivation, the total quantity of extractable raw materials was insufficient. As the preliminary experiment confirmed high homogeneity of the three extracts, one representative extract was chosen for primary metabolomics analysis, while the rest were applied to the isolation, purification and structural elucidation of natural monomer compounds. This scheme satisfies both the analysis of metabolic profiles and the preparation of bioactive monomers, maximizing the utilization of rare experimental materials.

#### 2.2.2. UPLC-ESI-QTRAP-MS/MS Analysis for MRM-Based Metabolite Profiling

MRM-based metabolite profiling was performed using a UPLC-ESI-QTRAP-MS/MS system consisting of an ExionLC™ AD UPLC system (SCIEX, Framingham, MA, USA) coupled to a QTRAP tandem mass spectrometer (SCIEX, Framingham, MA, USA). This platform was used for widely targeted metabolite detection based on predefined MRM transitions.

Chromatographic separation was achieved on an Agilent SB-C18 column (1.8 μm, 2.1 × 100 mm). Solvent A was acetonitrile (Honeywell Research Chemicals, Muskegon, MI, USA) containing 0.1% formic acid (J&K Scientific Ltd., Beijing, China), and solvent B was water containing 0.1% formic acid. The gradient program was as follows: 5–95% A from 0 to 9 min, 95% A from 9 to 10 min, 95–5% A from 10 to 10.1 min, and 5% A from 10.1 to 12.5 min. The column temperature, flow rate, and injection volume were set to 40 °C, 0.35 mL/min, and 2 μL, respectively. The column effluent was introduced directly into the ESI-QTRAP-MS/MS system.

The ESI source parameters were set as follows: ion-spray voltage, −4500 V in negative-ion mode and 5500 V in positive-ion mode; source temperature, 500 °C; curtain gas, 25 psi; ion source gas I, 50 psi; and ion source gas II, 60 psi. Collision-activated dissociation (CAD) was set to high. Nitrogen was used as the collision gas. MRM experiments were conducted in triple-quadrupole mode, and specific precursor/product ion transitions corresponding to the eluting metabolites were monitored. The declustering potential (DP) and collision energy (CE) values for each MRM transition were optimized individually.

#### 2.2.3. MRM-Based Metabolite Annotation and Data Processing

Metabolite annotation in the MRM dataset was performed by matching the detected signals against an in-house database containing retention times, precursor/product ion pairs, and optimized MS parameters. The annotation criteria included retention time (RT), Q1/Q3 ion transitions, and the corresponding DP and CE values. The RT tolerance was set to 0.2 min, and the Q1/Q3 transition tolerance was set according to the database settings. Data acquisition and processing were performed under consistent quality-control procedures throughout the analytical sequence.

Detected metabolites were classified into three confidence tiers according to the matching scores:

Tier 1: The metabolites in the sample match the database in terms of MS^1^, MS^2^, and RT.
Tier 2: The metabolites in the sample match the database in terms of both MS^1^ and MS^2^.
Tier 3: The metabolites in the sample match the database only in terms of MS^1^.

Metabolites classified as Tier 1 and Tier 2 all have a score greater than 0.5, while those at Tier 3 have a score lower than 0.5 and are not displayed by default.

The annotation method for non-targeted metabolites is as follows: The raw data files (.mzxml) are imported into the XCMS database search software (version 3.7.1), where simple filtering of parameters such as retention time and mass-to-charge ratio (*m*/*z*) is performed. Subsequently, peak alignment is carried out across different samples based on retention time deviation and mass deviation to improve annotation accuracy. Peak extraction is then performed according to the set parameters, including mass deviation, signal intensity deviation, signal-to-noise ratio (S/N), and minimum signal intensity. The target ions are then integrated, followed by molecular formula prediction and comparison with the database. Background ions are removed using blank samples, and the quantitative results are normalized using QC samples. Finally, the annotation results of the data are obtained.

Rigorous quality control (QC) measures were adopted throughout data acquisition and preprocessing for untargeted metabolomic analysis. Pooled QC samples, prepared by equally mixing all experimental samples, were regularly injected to stabilize the chromatographic and mass spectrometry system and monitor instrumental drift. Blank injections were performed for system flushing and background contamination subtraction. In data preprocessing, metabolites with a missing value ratio over 50% were eliminated, and residual missing values were imputed via the KNN algorithm. Features contaminated by background signals were removed, and QC-based normalization was applied to correct systematic drift. Only metabolites with a QC coefficient of variation (CV) < 30% were retained. Redundant metabolites were deduplicated based on InChIKey or English names, with priority given to reliable annotation tiers and high matching scores. Given the limited quantity of seed materials, the metabolomics results in this study were interpreted primarily as a qualitative chemical profile rather than absolute quantitative data.

Verification results of three groups of parallel extracts at the preliminary stage demonstrated that there were no significant differences in metabolic compositions among all groups of *C. appendiculata* seed samples, with favorable sample homogeneity. A single extract is capable of reflecting the basal metabolic background characteristics of seeds. This analysis constitutes exploratory qualitative profiling based on one representative extract.

#### 2.2.4. UPLC-Q-TOF-MS/MS Analysis for Untargeted MS/MS Acquisition and Secondary Metabolite Characterization

The seed extracts of *C. appendiculata* were further analyzed using a Waters Xevo G2-S QTOF UPLC-MS/MS system controlled by MassLynx 4.1 software. This high-resolution MS/MS dataset was used for secondary metabolite characterization and molecular networking analysis.

Chromatographic separation was performed using a Waters ACQUITY UPLC-PDA system equipped with a C18 column (1.7 μm, 2.1 × 100 mm; Waters, USA). The UV detection range was set to 200–400 nm, and the column temperature was maintained at 40 °C. The mobile phase consisted of solvent A, acetonitrile containing 0.1% formic acid, and solvent B, water containing 0.1% formic acid. The gradient program was as follows: 5% A from 0 to 26 min, 5–80% A from 26 to 30 min, 80–100% A from 30 to 30.1 min, and 100–5% A from 30.1 to 33 min, followed by 5% A for column re-equilibration, at a flow rate of 0.3 mL/min.

Leucine-enkephalin (Shanghai Yuanye Bio-Technology Co., Ltd., Shanghai, China) at 200 ng/mL was used as the lock-mass standard with the reference ion [M−H]^−^ at *m*/*z* 554.2615 and was infused at a flow rate of 10 μL/min. MS/MS data were acquired in FAST DDA mode with a full MS scan range of *m*/*z* 50–1500 in negative-ion mode. The desolvation gas flow was 900 L/h, the desolvation temperature was 450 °C, and the source temperature was 100 °C. The sample cone voltage was 50 V, the capillary voltage was 2.0 kV, the low collision energy was 4.0 eV, and the high-energy collision ramp was 20–40 eV. Data were processed using MassLynx 4.1.

### 2.3. Molecular Networking

The high-resolution UPLC-Q-TOF-MS/MS data were used for molecular networking analysis. Raw MS/MS data were converted from .raw format to .msp and .csv formats using Progenesis QI and uploaded to MassIVE for molecular networking analysis on the GNPS platform. The precursor-ion and fragment-ion mass tolerances were both set to 0.02 Da. Molecular networks were generated using the following parameters: minimum cluster size, 2; minimum number of matched fragment ions, 5; cosine score threshold, 0.65; and Network TopK, 10. The resulting molecular networks were visualized using Cytoscape 3.9.0.

The molecular networking results were used to organize structurally related metabolites according to their MS/MS fragmentation similarity and to assist in the dereplication and characterization of major secondary metabolite classes in *C. appendiculata* seeds. The network data and parameters are publicly accessible at https://gnps.ucsd.edu/ProteoSAFe/status.jsp?task=da4be5e56a634775bd68e04fe67c8fa9 (accessed on 20 March 2026).

### 2.4. Purification of Compounds

Purification of *C. appendiculata* seed extracts was carried out based on preliminary screening results obtained from UPLC-Q-TOF-MS/MS molecular networking clustering, with the scarcity of sample materials and isolation feasibility taken into consideration.

The EtOAc extract (9.0 g) was fractionated on an ODS (Qingdao Haiyang Chemical Co., Ltd., Qingdao, China) column eluted with a stepwise MeOH-H_2_O gradient (10–100%) to yield six major fractions (Fr.1–Fr.6). Compound **18** (3.2 mg, t_R_ = 25.6 min) was isolated from Fr.1 (24.1 mg) by semi-preparative HPLC (0–32 min, 26% MeOH; 32.1–40 min, 100% MeOH; 40.1 min, 25% MeOH). Compound **15** (7.1 mg, t_R_ = 22.4 min) was isolated from Fr.2 (134.0 mg) by semi-preparative HPLC (0–25 min, 16% MeOH). Fr.3 (120.5 mg) was separated by Sephadex LH-20 (Pharmacia, Uppsala, Sweden) chromatography (MeOH) to afford Fr.3.1 (55.6 mg) and Fr.3.2 (8.6 mg). Compound **16** (3.3 mg, t_R_ = 29.3 min) was purified from Fr.3.1 (55.6 mg) by semi-preparative HPLC (0–20 min, 23–45% MeOH; 20–30 min, 45% MeOH), and compound **17** (1.1 mg, t_R_ = 38.6 min) was obtained from Fr.3.2 (8.6 mg) by semi-preparative HPLC (0–40 min, 30% MeOH).

Fr.4 (820.1 mg) was separated by Sephadex LH-20 chromatography (MeOH) to afford three fractions (Fr.4.1–Fr.4.3). Compound **8** (4.1 mg) was purified from Fr.4.1 (32.9 mg) by semi-preparative HPLC (35% MeOH-H_2_O). Fr.4.2 (754.0 mg) was subjected to normal-phase chromatography (petroleum ether-acetone, Beijing Chemical Reagent Company, Beijing, China) to obtain Fr.4.2.1 and Fr.4.2.2. Compound **14** (2.7 mg, t_R_ = 33.2 min) was purified from Fr.4.2.1 (16.5 mg) by semi-preparative HPLC. Compounds **6** (1.5 mg, t_R_ = 22.2 min) and **13** (1.5 mg, t_R_ = 30.2 min) were isolated from Fr.4.2.2 (43.2 mg) by semi-preparative HPLC (0–2 min, 21–22.5% ACN-H_2_O; 2–21 min, 22.5% ACN-H_2_O; 21.1–29 min, 100% ACN-H_2_O; 29.1 min, 21% ACN-H_2_O).

Fr.5 (306.1 mg) was separated by Sephadex LH-20 chromatography (MeOH) to afford three fractions (Fr.5.1–Fr.5.3). Compound **3** (2.3 mg, t_R_ = 30.2 min) was purified from Fr.5.1 (40.2 mg) by semi-preparative HPLC (27% ACN-H_2_O). Fr.6 (170.6 mg) was subjected to Sephadex LH-20 chromatography (MeOH) to yield three fractions (Fr.6.1–Fr.6.3). Compounds **4** (5.6 mg, t_R_ = 32.0 min) and 1 (3.0 mg, t_R_ = 34.1 min) were obtained from Fr.6.3 (65.2 mg) by semi-preparative HPLC (43% MeOH-H_2_O).

The *n*-BuOH extract (5.0 g) was fractionated on an ODS column eluted with a stepwise MeOH-H_2_O gradient (10–100%) to yield eight major fractions (Fr.1–Fr.8). Compound **7** (7.6 mg, t_R_ = 25.2 min) was purified from Fr.3 by semi-preparative HPLC (0–30 min, 24% MeOH-H_2_O). Compounds **2** (5.4 mg, t_R_ = 13.8 min) and **12** (2.4 mg, t_R_ = 29.9 min) were isolated from Fr.4 by semi-preparative HPLC (0–30 min, 14% ACN-H_2_O). Compound **19** (9.6 mg, t_R_ = 21.2 min) was purified from Fr.5 by semi-preparative HPLC (0–40 min, 25% MeOH-H_2_O). Compounds **10** (15.9 mg, t_R_ = 20.3 min) and **11** (18.3 mg, t_R_ = 22.4 min) were obtained from Fr.7 by semi-preparative HPLC (0–40 min, 35% MeOH-H_2_O). Fr.8 (98.3 mg) was separated by Sephadex LH-20 chromatography (MeOH) to afford five fractions (Fr.8.1–Fr.8.5). Compound **6** (3.1 mg, t_R_ = 27.9 min) was purified from Fr.8.3 by semi-preparative HPLC (0–35 min, 32% MeOH-H_2_O). Compound **5** (4.0 mg, t_R_ = 18.0 min) was obtained from Fr.8.5 by semi-preparative HPLC (0–20 min, 20–40% MeOH-H_2_O). Compound **9** (2.2 mg, t_R_ = 23.5 min) was purified from Fr.9 (157.4 mg) by semi-preparative HPLC (0–30 min, 30–50% MeOH-H_2_O).

*Cannabisin S* (**1**): [*α*${]}_{D}^{25}$ −7.0 (*c* 0.1, MeOH); UV (MeOH) *λ*_max_ (log *ε*) 205 (4.10), 288 (3.89), 319 (3.89) nm; IR (neat) *ν*_max_ 3300, 2938, 1657, 1596, 1515 and 1257 cm^−1^; ^1^H-NMR (500 MHz, CD_3_OD) and ^13^C NMR (125 MHz, CD_3_OD), Table 1; (-)-HR-ESI-MS: *m*/*z* 639.2412 [M−H]^−^ (calcd. for C_36_H_35_N_2_O_9_, 639.2415).

4-*O*-methoxybenzoyl-*β*-glucopyranosyl-(1→6)-*β*-glucopyranoside (**2**): [*α*${]}_{D}^{25}$ −61.99 (*c* 0.1, MeOH); UV (MeOH) *λ*_max_ (log *ε*) 206 (4.02), 250 (4.01) nm; IR (neat) *ν*_max_ 3363, 2888, 1713, 1606, 1285and 1245 cm^−1^; ^1^H-NMR (500 MHz, DMSO-*d*_6_) and ^13^C NMR (125 MHz, CDCl_3_), Table 1; (-)-HR-ESI-MS: *m*/*z* 475.1528 [M−H]^−^ (calcd. for C_20_H_29_O_13_, 475.1530).

### 2.5. Growth-Promoting Activity and Inhibition Activity Test

#### 2.5.1. Growth-Promoting Activity Test on *C. disseminatus*

To test growth-promoting activity, several primary and secondary metabolites were selected. All test compounds were dissolved with DMSO (Shanghai Macklin Biochemical Technology Co., Ltd., Shanghai, China, analytical reagent) or dd H_2_O (double-distilled water, A.S. Watson^TM^ Ltd., Hong Kong, China, mass spectrometry purity). The prepared solutions were added into water agar media (consisting of 20.0 g agar powder dissolved in 1 L distilled water, final concentration 20 g/L) to obtain final concentrations of 0.1, 1, 10 and 100 µM. The pH values of medium were measured to eliminate the impacts of pH variation on activity assays. The prepared medium was poured into microbial Petri dishes. After solidification, pre-cultured *C. disseminatus* mycelial plugs were placed at the center of each plate using a sterile cork borer. All plates were incubated at a constant temperature of 25 °C in the dark for 7 days, after which fungal growth was quantified. Notably, five biological replicates were set for each concentration, and a negative control group containing only the compound solvent was included. The measured index in this study is hyphal length, which is defined as the diameter of the fungal colony. The colony diameter crossing the center of the central mycelial plug was measured. *C. disseminatus* was provided by Chongqing Institute of Medicinal Plant Cultivation.

#### 2.5.2. Growth Inhibition Activity Test on *F. oxysporum*

*F. oxysporum* is a broad-spectrum pathogenic fungus that causes wilt and root diseases in numerous medicinal and ornamental orchid plants [11,12]. Considering that this fungus represents a major pathogenic fungus threatening orchid species, *F. oxysporum* was selected as the test strain to investigate the inhibitory effects of secondary metabolites against its growth. *F. oxysporum* was provided by Chongqing Institute of Medicinal Plant Cultivation. The methods were the same as in the preceding subsection.

#### 2.5.3. Statistical Analysis

Statistical analysis for the growth-promoting and antifungal activities of isolated compounds was performed with GraphPad Prism 9.5. Since all datasets failed the normality test, non-parametric statistics were applied. The overall difference among groups was first evaluated by the Kruskal–Wallis test, followed by Dunn’s multiple comparisons test with Bonferroni correction for pairwise comparisons to control the family-wise error rate. Five independent biological replicates (*n* = 5) were set for each treatment rather than technical replicates. Significance levels were set at *p* < 0.05 (*), *p* < 0.01 (**), *p* < 0.001 (***), and *p* < 0.0001 (****). All data are expressed as mean ± standard deviation (SD).

## 3. Results and Discussion

### 3.1. Metabolomic Analysis of C. appendiculata Seeds

**Figure 1:**
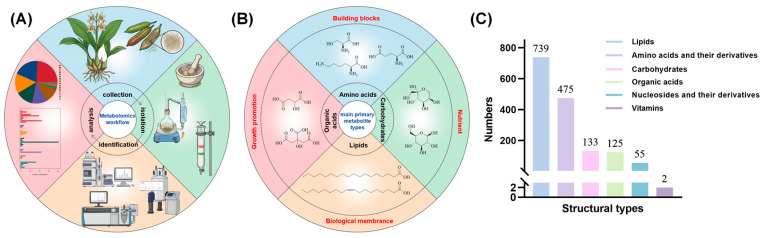
Metabolite analysis process (**A**), main primary metabolite types from *C. appendiculata* seeds (**B**) and numbers of the primary metabolite types (**C**).

Metabolomic analysis of *C. appendiculata* seeds annotated 1529 primary metabolites. These metabolites were mainly classified as lipids (739), amino acids and their derivatives (475), carbohydrates (133), organic acids (125), nucleosides and their derivatives (55), and vitamins (2) (Figure 1).

The lipids detected in *C. appendiculata* seeds comprised diverse structural types, including fatty acyls, prenol lipids, steroids and their derivatives, and glycerides (see supporting data for details). Among these, stearic acid, oleic acid, linoleic acid, and sitosterol were putatively annotated. Ma et al. [13] reported that AM symbiosis specifically upregulates genes involved in fatty acid synthesis and transport, thereby increasing fatty acid contents in plant roots and leaves, particularly oleic acid (18:1) and linoleic acid (18:2). Fatty acids serve as major carbon sources transferred from host plants to AM fungi, representing a conserved physiological feature of mycorrhizal symbiosis [14]. In plants, desaturation of stearic acid (18:0) to oleic acid (18:1) is a key step in fatty acid biosynthesis. Oleic acid can activate plant defense responses against pathogens by regulating nitric oxide synthesis and facilitate seed germination [15,16]. In addition, exogenous *β*-sitosterol has been shown to promote plant cell growth [17]. Relevant studies have confirmed that in the symbiotic relationship of orchid seeds, *Gymnadenia conopsea* seeds synthesize and secrete fatty acids, which function as carbon sources to support the growth of symbiotic fungal mycelia [18]. The lipids identified in *C. appendiculata* seeds may exert similar biological functions.

Metabolomic analysis also showed that amino acids occur in *C. appendiculata* seeds, including glycine, serine, lysine, and glutamic acid. Amino acids are efficient organic nitrogen sources that can be directly absorbed and utilized by plants. After uptake, they rapidly participate in protein synthesis and metabolism to support robust plant growth. Apart from their basic nutritional functions, certain amino acids also function as signaling molecules to regulate symbiotic fungal colonization and mediate plant defense responses against various environmental stresses. Yang et al. [19] found that glycine in banana root exudates enhanced the rhizosphere colonization ability of the beneficial bacterium *Bacillus velezensis* LG14-3 and contributed to the suppression of pathogen infection. Tao et al. [20] reported that serine secreted by plant roots acts as a key signal that triggers *Bacillus* spore germination and works synergistically with ornithine, isoleucine, valine, and alanine to enhance rhizosphere colonization and plant growth promotion. Huang and Fang [21] found that lysine promoted rhizome proliferation in *Cymbidium goeringii* without inducing browning. Furthermore, glutamic acid participates in multiple physiological processes, including seed germination, seedling establishment, plant growth, development, and senescence [22]. These findings suggest that amino acids may contribute to the establishment of symbiosis between *C. appendiculata* seeds and *C. disseminatus*.

The carbohydrates detected in *C. appendiculata* seeds mainly included monosaccharides, such as glucose, fructose, and mannose, and disaccharides, such as sucrose and trehalose. Ponert et al. [23] showed that soluble carbohydrates, including glucose, fructose, sucrose, and trehalose, promote protocorm formation and growth in orchids. Trehalose can be hydrolyzed to glucose at the mycorrhizal interface, thereby providing carbon and energy for orchid plants. Trehalose also plays an important role in AM symbiosis and contributes to plant tolerance to abiotic stress [24]. In addition, plant-secreted monosaccharides (e.g., fructose and mannose) and disaccharides (e.g., sucrose) can function as chemical inducers that attract specific microorganisms to roots and promote the establishment of beneficial symbiotic relationships [25]. Thus, in the *C. appendiculata*–*C. disseminatus* system, carbohydrates may serve not only as nutrients supporting fungal growth and seed germination but also as potential signaling molecules involved in partner recognition.

Organic acids and nucleosides, including citric acid, malic acid, uridine, adenosine, and 2′-deoxyadenosine, were also detected in *C. appendiculata* seeds. Plant-derived organic acids are important drivers of rhizosphere bacterial activity [26]. Citric acid and malic acid can selectively recruit beneficial soil microorganisms, upregulate genes related to bacterial chemotaxis and biofilm formation, and promote plant growth; conversely, colonization by beneficial bacteria can further enhance organic acid secretion from plant roots [27,28]. Uridine, adenosine, and 2′-deoxyadenosine are common components of plant root exudates and can act as chemoattractants that recruit beneficial microorganisms and regulate rhizosphere microbial community assembly [29]. This suggests that the organic acids and nucleosides in the seeds of *C. appendiculata* potentially function as nutrients or signaling molecules for the growth of symbiotic fungi.

Overall, metabolomic profiling provided a comprehensive overview of the primary metabolite composition of *C. appendiculata* seeds and established a foundation for identifying potential nutrients and signaling molecules involved in the *C. appendiculata*–*C. disseminatus* symbiotic system.

### 3.2. Molecular Networking Analysis

**Figure 2:**
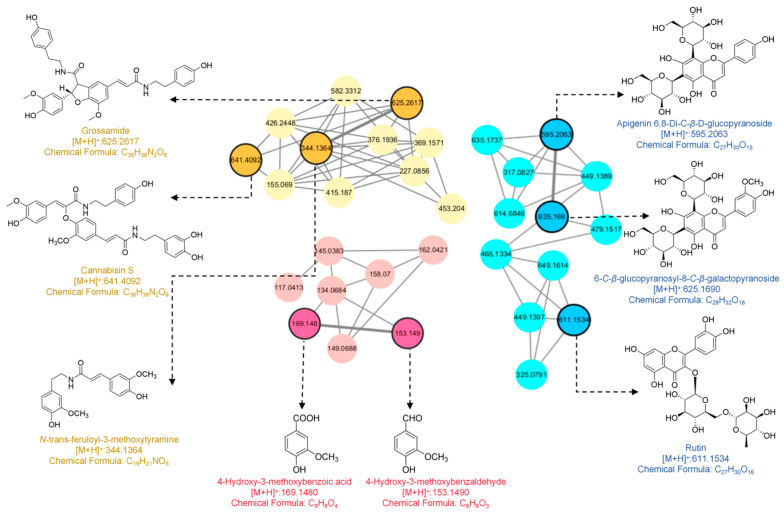
Molecular networking clusters of *C. appendiculata* seed extract.

To explore the secondary metabolites of *C. appendiculata* seeds, the crude extract was analyzed by UPLC-Q-TOF-MS/MS, and a molecular network was constructed on the GNPS platform to annotate and cluster structurally related metabolites. Based on MS/MS spectral similarity, three major clusters were identified and assigned as lignanamides (cluster I), flavonoids (cluster II), and phenolic acids (cluster III) (Figure 2).

In cluster I, grossamide (**4**), a representative lignanamide, was annotated using the GNPS library, consistent with its molecular formula (C_36_H_36_N_2_O_8_) and diagnostic MS/MS fragment ions at *m*/*z* 625 and 462. Subsequently, a trimeric lignanamide, cannabisin S (**1**), was tentatively characterized with a molecular formula of C_36_H_38_N_2_O_9_ and fragment ions at *m*/*z* 643, 460, and 314. The ion at *m*/*z* 460 was attributed to the loss of a 4-(2-aminoethyl)phenol moiety and one H_2_O molecule. Another node exhibited a molecular ion at *m*/*z* 344 and a characteristic fragment ion at *m*/*z* 177, suggesting the loss of a 3-methoxytyramine unit to generate a trans-feruloyl moiety (*m*/*z* 177). Accordingly, this compound was tentatively identified as *N*-trans-feruloyl-3-methoxytyramine (**3**) by comparing its molecular formula and MS/MS fragmentation pattern with data from the literature. In cluster II, three flavonoids were matched against the GNPS library and showed characteristic fragment ions at *m*/*z* 153 and 123, corresponding to the flavonoid core. Based on their MS/MS fragmentation patterns, these compounds were tentatively identified as rutin (**9**), apigenin 6,8-di-*C*-*β*-D-glucopyranoside (**10**), and 6-*C*-*β*-glucopyranosyl-8-*C*-*β*-galactopyranoside (**11**). In cluster III, two phenolic acids were annotated using the GNPS library, both displaying a common fragment ion at *m*/*z* 137 corresponding to a guaiacyl unit. These compounds were identified as 4-hydroxy-3-methoxybenzoic acid (**16**) and 4-hydroxy-3-methoxybenzaldehyde (**17**).

**Figure 3:**
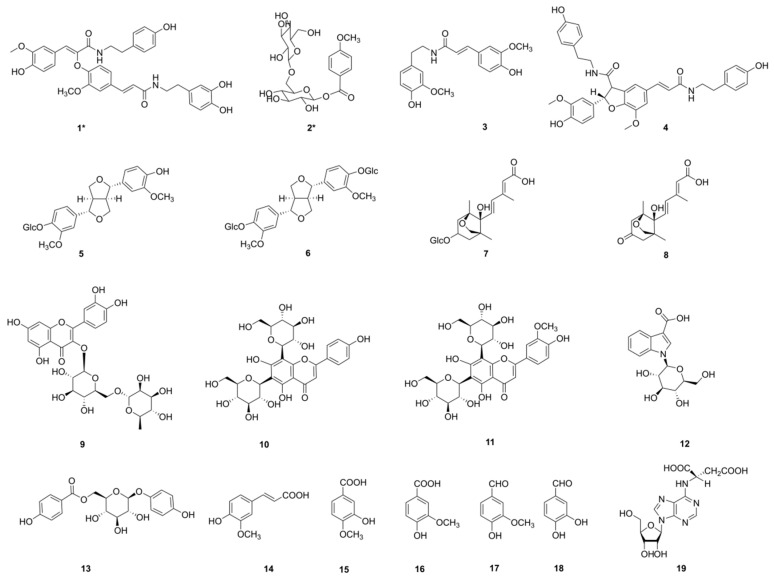
The structures of compounds **1**–**19** identified in seed extracts.

To verify these preliminarily identified compounds, systematic separation was carried out on the crude extract of the *C. appendiculata* seeds. Nineteen compounds were isolated, including eight compounds annotated in the molecular network and two new analogs (**1** and **2**) (Figure 3).

### 3.3. Structural Elucidation

**Figure 4:**
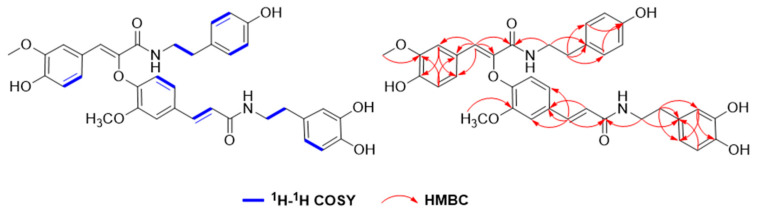
Key 2D NMR correlations of compound **1**.

The molecular formula of compound **1** was determined as C_36_H_36_N_2_O_9_ based on HR-ESI-MS data (*m*/*z* 639.2412, [M−H]^−^). Detailed interpretation of the ^1^H NMR, ^13^C NMR, and HSQC spectra revealed two methoxy groups, four methylene groups, fourteen aromatic protons, and fourteen quaternary carbons. In the ^1^H NMR spectrum, four ortho-coupled aromatic protons at *δ*_H_ 6.84 (H-2″, H-6″) and 6.59 (H-3″, H-5″) indicated the presence of one para-disubstituted benzene ring. Nine additional aromatic protons were assigned to three 1,3,4-trisubstituted benzene rings: *δ*_H_ 7.04 (1H, dd, *J* = 8.5, 2.0 Hz, H-6), 6.73 (1H, d, *J* = 8.5 Hz, H-5), and 7.29 (1H, d, *J* = 2.0 Hz, H-2); *δ*_H_ 7.02 (1H, dd, *J* = 8.0, 2.0 Hz, H-6′), 6.71 (1H, d, *J* = 8.0 Hz, H-5′), and 7.24 (1H, d, *J* = 2.0 Hz, H-2′); and *δ*_H_ 6.55 (1H, dd, *J* = 8.0, 2.0 Hz, H-6″), 6.69 (1H, d, *J* = 8.0 Hz, H-5″), and 6.67 (1H, d, *J* = 2.0 Hz, H-2″). Two pairs of methylene protons at *δ*_H_ 3.45 (2H, t, *J* = 7.0 Hz, H-8″)/2.64 (2H, t, *J* = 7.0 Hz, H-7″) and *δ*_H_ 3.47 (2H, t, *J* = 7.0 Hz, H-8‴)/2.70 (2H, t, *J* = 7.0 Hz, H-7‴) suggested the presence of two NHCH_2_CH_2_ units. A pair of trans-olefinic protons at *δ*_H_ 7.46 (1H, d, *J* = 15.5 Hz, H-7′) and 6.50 (1H, d, *J* = 15.5 Hz, H-8′), together with a conjugated olefinic singlet at *δ*_H_ 7.24 (1H, s, H-7), indicated two double-bond systems. The planar structure was established by analysis of the ^1^H-^1^H COSY and HMBC correlations. Compound **1** was assigned as a lignanamide analog containing two *N*-trans-caffeoyltyramine units, which were connected through an oxygen bridge between C-8 and C-4′, as supported by the key HMBC correlation from H-7 to C-4′. Database searching in SciFinder confirmed that compound **1** is a new lignanamide analog, named cannabisin S (Figure 4 and Figures S2–S8).

**Figure 5:**
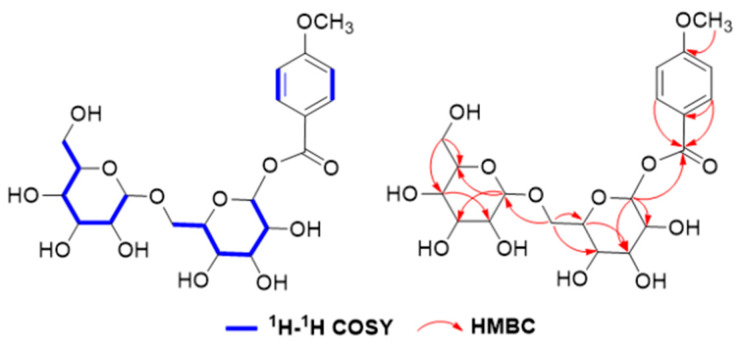
Key 2D NMR correlations of compound **2**.

The molecular formula of compound **2** was determined to be C_20_H_28_O_13_ with seven degrees of unsaturation based on its (-)-HR-ESI-MS data. Analysis of the ^1^H NMR, ^13^C NMR, and HSQC spectra revealed one methoxy group, two methylene groups, and fourteen methine groups, including four aromatic protons and ten oxygenated methine protons. The molecule also contained quaternary carbons, including one ester carbonyl carbon and aromatic quaternary carbons. These data suggested that compound **2** was a phenolic acid glycoside structurally related to 4-*O*-methoxybenzoyl-*β*-glucopyranosyl-(1→6)-*β*-glucopyranoside. In the ^1^H NMR spectrum, four ortho-coupled protons at *δ*_H_ 7.91 (H-2″/H-6″) and *δ*_H_ 7.18 (H-3″/H-5″) indicated a para-disubstituted benzene ring. The downfield shifts in H-2″ and H-6″ suggested attachment of an electron-withdrawing group, and the carbonyl carbon signal at *δ*_C_ 165.9 further supported the presence of an ester moiety. The methoxy signal at *δ*_H_ 3.82 (3H) indicated a methoxy substituent at the para-position relative to the ester group. The ^1^H-^1^H COSY spectrum established four spin systems: C-2″/C-3″, C-5″/C-6″, C-1/C-2/C-3/C-4/C-5, and C-1′/C-2′/C-3′/C-4′/C-5′. A key HMBC correlation from H-1 to C-7″ demonstrated that the sugar unit was linked to C-7″ through an ester bond. Additional HMBC correlations from H-1 to C-2/C-3 and from H-6 to C-4/C-5 supported the glucose assignment. The HMBC correlation from H-6 to C-1′ confirmed that the oxygenated methylene of the first glucose was connected to the anomeric carbon of the second glucose, forming a disaccharide glycoside. Limited by insufficient sample quantity, determination of absolute configuration was not performed in this study. Thus, the planar structure of compound **2** was established. SciFinder searching confirmed compound **2** to be a new phenolic acid glycoside, named 4-*O*-methoxybenzoyl-*β*-glucopyranosyl-(1→6)-*β*-glucopyranoside (Figure 5 and Figures S9–S15).

### 3.4. Growth-Promoting and Inhibitory Activity of Metabolites

To evaluate the ecological relevance of metabolites from *C. appendiculata* seeds, several primary and secondary metabolites were selected based on the structural categories of the isolated compounds, and their biological activities on the symbiotic fungus *C. disseminatus* and the plant pathogenic fungus *F. oxysporum* were separately evaluated.

The tested amino acids showed distinct effects on mycelial growth depending on their acid–base properties. The neutral amino acid L-serine promoted mycelial growth at a low concentration (0.1 μM), whereas basic L-lysine and acidic L-glutamate showed no growth-promoting activity and even exerted inhibitory effects. These results suggest that *C. disseminatus* may selectively respond to amino acids according to their acid–base characteristics. Acidic and basic amino acids can alter microenvironmental pH and disrupt intracellular homeostasis, thereby suppressing fungal growth [30]. By contrast, neutral amino acids may be more readily utilized by this fungus as exogenous nutrients or chemical cues.

Fructose, sucrose, and mannose did not significantly promote fungal growth compared with the control, whereas glucose and trehalose exerted positive effects at a high concentration (100 μM). 

These results indicate that *C. disseminatus* exhibits preferences for different sugars. Although fructose, sucrose and mannose are common plant metabolites, *C. disseminatus* preferentially utilizes glucose and trehalose under the tested conditions. The growth-promoting effects of glucose and trehalose at high concentrations suggest that glucose may serve as a primary utilizable carbon source, whereas trehalose may contribute indirectly after hydrolysis to glucose.

**Figure 6:**
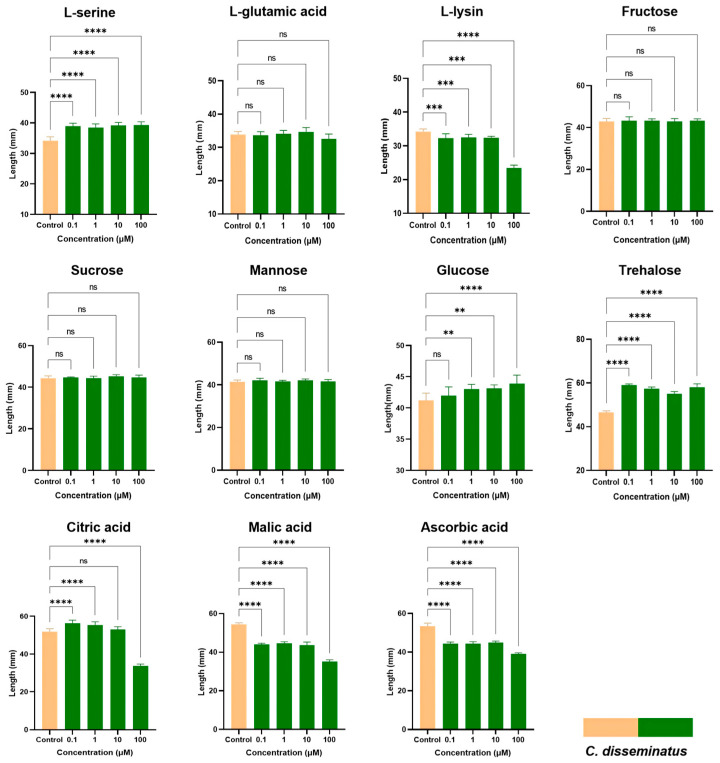
Growth-promoting activity of primary metabolites of *C. appendiculata* seed on the fungus *C. disseminatus*. Dunn’s test was utilized to evaluate the statistical significance of the disparity between the treated and control groups, with the corresponding *p* values presented. *p* > 0.05 (ns), *p* < 0.01 (**), *p* < 0.001 (***), *p* < 0.0001 (****). All groups were set up with five biological replicates (*n* = 5), and the data are presented as mean ± SD.

Citric acid promoted mycelial growth at a low concentration (0.1 μM), with activity increasing initially and then declining as the concentration increased, whereas malic acid and ascorbic acid showed inhibitory effects (Figure 6). At low concentrations, citric acid may support fungal growth by entering the tricarboxylic acid (TCA) cycle as a central energy-metabolism intermediate. At higher concentrations, however, citric acid may inhibit growth by disturbing pH balance or imposing osmotic stress, resulting in a typical concentration-dependent dual effect [31].

**Figure 7:**
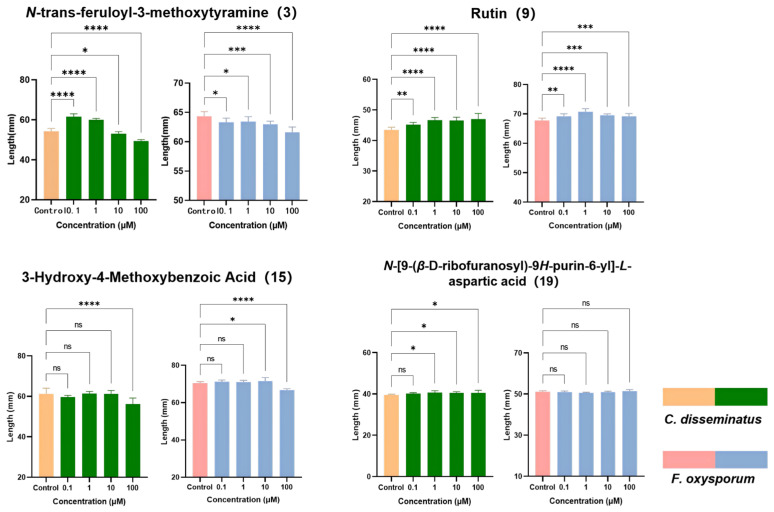
Biological activities of compounds **3**, **9**, **15** and **19** on the mycorrhizal fungus *C. disseminatus* and the pathogenic fungus *F. oxysporum*. Dunn’s test was utilized to evaluate the statistical significance of the disparity between the treated and control groups, with the corresponding *p* values presented. *p* > 0.05 (ns), *p* < 0.05 (*), *p* < 0.01 (**), *p* < 0.001 (***), *p* < 0.0001 (****). All groups were set up with five biological replicates (*n* = 5), and the data are presented as mean ± SD.

Lignanamide compound **3** promoted the growth of *C. disseminatus* at a low concentration (0.1 μM) and inhibited *F. oxysporum* in a concentration-dependent manner. Flavonoid compound **9** showed moderate growth-promoting activity toward both *C. disseminatus* and *F. oxysporum*. Phenolic acid compound **15** inhibited both *C. disseminatus* and *F. oxysporum* at a high concentration (100 μM). Nucleoside compound **19** did not show significant growth-promoting or inhibitory activity against either fungus at a low concentration (0.1 μM) (Figure 7). Among the tested secondary metabolites, lignanamides exhibited potential dual ecological functions by promoting the growth of beneficial fungi at low concentrations while suppressing the propagation of pathogenic fungi [32].

Therefore, neutral amino acids, glucose, citric acid, and lignanamides merit further investigation as potential bioactive molecules.

## 4. Conclusions

This study systematically investigated primary and secondary metabolites from *C. appendiculata* seeds by integrating metabolomic profiling, UPLC-Q-TOF-MS/MS analysis and molecular networking. A total of 1529 primary metabolites were annotated from the seed extracts and classified mainly as lipids (739), amino acids and their derivatives (475), carbohydrates (133), organic acids (125), nucleosides and their derivatives (55), and vitamins (2). Amino acids with different acid–base properties showed distinct effects on the mycelial growth of *C. disseminatus*, with the neutral amino acid L-serine exhibiting the most favorable growth-promoting effect among the tested amino acids. In carbohydrate utilization assays, *C. disseminatus* responded preferentially to glucose and trehalose. Citric acid produced a dual effect characterized by growth promotion at low concentration and inhibition at higher concentrations. Through systematic isolation of secondary metabolites from *C. appendiculata* seeds, two new compounds (**1** and **2**) and seventeen known compounds (**3**–**19**) were obtained. Among them, lignanamide compound **3** exhibited dual ecological activity, promoting the growth of *C. disseminatus* while inhibiting *F. oxysporum*.

As a typical plant–microbe symbiotic relationship, the seeds of *C. appendiculata* require symbiosis with *C. disseminatus* to initiate germination. Based on metabolomic analysis and the integrated strategy of UPLC-Q-TOF-MS combined with molecular networking, this work clarified the chemical composition of *C. appendiculata* seeds and identified candidate metabolites that may contribute to fungal growth promotion and pathogen suppression. Although endogenous metabolites are not necessarily secreted extracellularly, this study still identifies potential bioactive compounds in orchid seeds that modulate the proliferation of symbiotic and pathogenic fungi from a phytochemical perspective, laying a foundation for subsequent mechanistic research on chemical communication between seeds and fungi.

## Figures and Tables

**Table 1:** ^1^H and ^13^C NMR data of compound **1** in CD_3_OD and compound **2** in DMSO-*d*_6_.

Position	1		2	
	*δ*_H_ ^a^ (mult, *J* in Hz)	*δ*_C_ ^b^, Type	*δ*_H_ ^a^ (mult, *J* in Hz)	*δ*_C_ ^b^, Type
1		125.5, C	4.94, m	100.0, CH
2	7.29, d, (2.0)	113.7, CH	3.28, m	73.2, CH
3		148.9, C	3.28, m	76.4, CH
4		149.5, C	3.14, m	69.8, CH
5	6.73, d, (8.5)	1163, CH	3.64, m	75.9, CH
6	7.04, dd, (8.5, 2.0)	126.3, CH	4.01, dd, (12.0, 1.5) 3.57, dd, (12.0, 7.0)	68.7, CH_2_
7	7.24, s	125.3, CH		
8		141.5, C		
9		165.5, C		
1′		131.9, C	4.19, d, (7.5)	103.5, CH
2′	7.02, dd, (8.5, 2.0)	112.5, CH	2.97, m	73.6, CH
3′		150.5, C	3.10, m	76.7, CH
4′		147.5, C	3.03, m	70.2, CH
5′	6.71, d, (8.5)	115.2, CH	3.03, m	76.8, CH
6′	7.24, d, (2.0)	122.3, CH	3.67, m 3.42, dt, (11.0, 5.0)	61.1, CH_2_
7′	7.46, d, (15.5)	141.1, CH		
8′	6.50, d, (15.5)	121.0, CH		
9′		168.8, C		
1″		130.8, C		123.0, C
2″	6.84, m	130.7, CH	7.91, m	131.1, CH
3″	6.59, m	116.3, CH	7.18, m	116.3, CH
4″		156.8, C		165.9, C
5″	6.59, m	116.3, CH	7.18, m	116.3, CH
6″	6.84, m	130.7, CH	7.91, m	131.1, CH
7″	2.64, t, (7.0)	35.6, CH		161.1, C
8″	3.45, t, (7.0)	42.2, CH		
1‴		132.0, C		
2‴	6.67, d, (2.0)	116.9, CH		
3‴		146.3, C		
4‴		144.8, C		
5‴	6.69, d, (8.5)	116.4, CH		
6‴	6.55, dd, (8.5, 2.0)	121.0, CH		
7‴	2.70, t, (7.0)	36.0, CH		
8‴	3.47, t, (7.0)	42.6, CH		
2-OH			5.40, d, (3.5)	
3-OH			5.20, d, (5.5)	
4-OH			5.18, d, (5.5)	
2′-OH			4.97, d, (3.5)	
3′-OH			4.95, m	
4′-OH			4.90, d, (4.0)	
6′-OH			4.48, t, (6.0)	
3-OCH_3_	3.66, s	56.0, CH_3_		
3′-OCH_3_	3.93, s	56.4, CH_3_		
4″-OCH_3_			3.82, s	51.9, CH_3_

^a^ Recorded at 500 MHz, ^b^ recorded at 125 MHz.

## Data Availability

The original contributions presented in this study are included in the article/Supplementary Material. Further inquiries can be directed to the corresponding authors.

## Supplementary Materials

The following supporting information can be downloaded at: https://www.mdpi.com/article/10.3390/plants15152399/s1, Figure S1. HPLC chromatograms for reproducibility assessment. Figure S2. ^1^H NMR spectrum (500 MHz) of compound **1** in CD_3_OD. Figure S3. ^13^C NMR spectrum (125 MHz) of compound **1** in CD_3_OD. Figure S4. ^1^H-^1^H COSY spectrum (500 MHz) of compound **1** in CD_3_OD. Figure S5. HSQC spectrum (500 MHz) of compound **1** in CD_3_OD. Figure S6. HMBC spectrum (500 MHz) of compound **1** in CD_3_OD. Figure S7. IR spectrum of compound **1**. Figure S8. UV spectrum of compound **1** in MeOH. Figure S9. ^1^H NMR spectrum (500 MHz) of compound **2** in DMSO-*d*_6_. Figure S10. ^13^C NMR spectrum (125 MHz) of compound **2** in DMSO-*d*_6_. Figure S11. ^1^H-^1^H COSY spectrum (500 MHz) of compound **2** in DMSO-*d*_6_. Figure S12. HSQC spectrum (500 MHz) of compound **2** in DMSO-*d*_6_. Figure S13. HMBC spectrum (500 MHz) of compound **2** in DMSO-*d*_6_. Figure S14. IR spectrum of compound **2**. Figure S15. UV spectrum of compound **2** in MeOH. Table S1. Antioxidant activities of compounds **3**, **9**, **14** and **18.** Table S2. HPLC chromatograms data. Table S3. Metabolites analysis of lipids. Table S4. Metabolites analysis of amino acids and derivatives. Table S5. Metabolites analysis of carbohydrates. Table S6. Metabolites analysis of organic acids. Table S7. Metabolites analysis of nucleosides and derivatives. Table S8. Metabolites analysis of vitamins. Table S9. Activity test data. Table S10. Compounds information; Refs. [33,34,35] are cited in supplementary materials.

## Abbreviations

The following abbreviations are used in this manuscript:

AM	Arbuscular Mycorrhizal
OMF	Orchid Mycorrhizal Fungi
LCOs	Lipo-Chitooligosaccharides
MRM	Multiple Reaction Monitoring
HPLC	High-Performance Liquid Chromatography
NMR	Nuclear Magnetic Resonance
CAD	Collision-Activated Dissociation
DP	Declustering Potential
CE	Collision Energy
RT	Retention time
DPPH	1,1-diphenyl-2-picrylhydrazyl
dd H_2_O	Double-Distilled Water
