# Supplementary material for: Identification of Bioactive Metabolites from Dust-like Seeds of Cremastra appendiculata via Metabolomics, UPLC-Q-TOF-MS/MS, and Molecular Networking
Source: Plants (Basel). 2026 Aug 5;15(15):2399. doi: 10.3390/plants15152399 (PMC13468472; doi:10.3390/plants15152399)
Supplement: Supplementary file 1 [file plants-15-02399-s001.zip › Supporting Information.pdf]

Supporting Information

Identification of Bioactive Metabolites from Dust-like Seeds of *Cremastra appendiculata* via Metabolomics, UPLC-Q-TOF-MS/MS, and Molecular Networking

Zhao Liu [1], Yuan Chen [1], Kai-Peng Liu [1], Ruo-Xuan Xu [1], Gang Ding [1,*] and Yan-Duo Wang [1,2,*]

1 State Key Laboratory of Bioactive Substance and Function of Natural Medicines, Institute of Medicinal Plant Development, Chinese Academy of Medical Sciences and Peking Union Medical College, Beijing 100193, China; s2024009050@student.pumc.edu.cn (Z.L.); 17732790202@163.com (Y.C.); s2025009055@student.pumc.edu.cn (K.-P.L.); 13589963816@163.com (R.-X.X.)

2 Department of Pharmacy, The First Affiliated Hospital of Army Medical University, Chongqing 400038, China

* Correspondence: gding@implad.ac.cn (G.D.); wangyanduo1115@163.com (Y.-D.W.)

Contents

Supplementary Methods 3

Supplementary Materials References 5

Supplementary Figures 6

Figure S1 HPLC chromatograms for reproducibility assessment 6

Figure S2 ^{1}H NMR spectrum (500 MHz) of compound **1** in CD_3OD 7

Figure S3 ^{13}C NMR spectrum (125 MHz) of compound **1** in CD_3OD 7

Figure S4 ^{1}H-^{1}H COSY spectrum (500 MHz) of compound **1** in CD_3OD 8

Figure S5 HSQC spectrum (500 MHz) of compound **1** in CD_3OD 8

Figure S6 HMBC spectrum (500 MHz) of compound **1** in CD_3OD 9

Figure S7 IR spectrum of compound **1** 9

Figure S8 UV spectrum of compound **1** in MeOH 10

Figure S9 ^{1}H NMR spectrum (500 MHz) of compound **2** in DMSO-d_6 10

Figure S10 ^{13}C NMR spectrum (125 MHz) of compound **2** in DMSO-d_6 11

Figure S11 ^{1}H-^{1}H COSY spectrum (500 MHz) of compound **2** in DMSO-d_6 11

Figure S12 HSQC spectrum (500 MHz) of compound **2** in DMSO-d_6 12

Figure S13 HMBC spectrum (500 MHz) of compound **2** in DMSO-d_6 12

Figure S14 IR spectrum of compound **2** 13

Figure S15 UV spectrum of compound **2** in MeOH 13

Supplemental Table 14

Table S1 Antioxidant activities of **3**, **9**, **14** and **18** 14

Supplementary Methods

2.6. Antioxidant Activities Assay

The seeds of *C. appendiculata* lack endosperm, and are exposed to strong light, drought and oxidative stress for a long time in the natural environment. Low levels of reactive oxygen species (ROS) contribute to the maintenance of seed dormancy, while high levels of ROS promote seed deterioration, and appropriate ROS levels facilitate the transition from the dormant seeds to germinated seeds [1]. The metabolites of lignanamides, flavonoids and phenolic acids contained in seeds have basic antioxidant capacity, which suggests that the endogenous secondary metabolites of seeds may mitigate oxidative injury by eliminating excess intracellular ROS such as hydroxyl radicals and hydrogen peroxide, thereby preserving cellular integrity and physiological viability. It lays a physiological foundation for the subsequent establishment of symbiotic interaction between seeds and symbiotic fungi. In addition, the seed germination process not only induces the synthesis of a large number of secondary metabolites, but also activates the intrinsic plant antioxidant defense system to resist various biotic and abiotic stresses. Previous investigations have documented that the contents of superoxide dismutase (SOD) and catalase (CAT) were higher than those of ungerminated seeds after the symbiotic fungi invaded the seeds to form protocorm, and the two types of antioxidant enzymes could alleviate the damage of oxidative damage to host cells [2]. Therefore, the endogenous antioxidant metabolites of seeds may cooperate with them to scavenge excessive ROS, thereby creating a favorable microenvironment for fungal infection and colonization. Based on the above reasons, we evaluated the antioxidant activity of the compounds isolated from the experiment.

The 1,1-diphenyl-2-picrylhydrazyl (DPPH) assay was applied to evaluate the in vitro antioxidant capacity of 19 isolated compounds[3]. Each test compound was dissolved in suitable solvents such as methanol or DMSO to prepare solutions with

serial concentration gradients. A volume of 100 μL sample solution of varying concentrations was mixed with 100 μL freshly prepared 0.1 mM DPPH ethanol solution in a 96-well plate. After 30 minutes of light-shielded reaction at room temperature, the absorbance was measured at 517 nm. Blank control consisting of pure solvent, negative control consisting of solvent and DPPH solution and positive control consisting of ascorbic acid (Beijing Chemical Reagent Company, Beijing, China) were set up in the experiment. The free radical scavenging rate was calculated according to the formula Eq.S1, where $A_{control}$ and A_{sample} represented the absorbance of the negative control group and sample group respectively.

$$\text{Scavenging rate} = \frac{A_{control} - A_{sample}}{A_{control}} \times 100\% \quad \text{(Eq.S1)}$$

The dose-response curve was fitted via nonlinear regression analysis adopting the Sigmoidal dose-response model with compound concentration as the abscissa and scavenging rate as the ordinate, and the half maximal inhibitory concentration IC_{50} was calculated. All experiments were performed in triplicate independently, and results were presented as mean ± standard deviation (SD).

Compounds **1**-**19** were evaluated for antioxidant activity using the DPPH radical-scavenging assay. Only compounds **3**, **9**, **14**, and **18** showed antioxidant activity, although their potency was lower than that of the positive control, ascorbic acid (Table S1).

Supplementary Materials References

1. Jhanji, S.;Goyal, E.; Chumber, M.; Kaur, G. Exploring fine tuning between phytohormones and ros signaling cascade in regulation of seed dormancy, germination and seedling development. *Plant Physiol. Biochem*. **2024**, *207*, 108352.
2. Huang, L.; Gao, Y. Mycorrhizal symbiosis between *Coprinellus disseminatus* and *Cremastra appendiculata*, insights from gene expression. *Sci Rep* **2026**, *16*, 3705.
3. Brand-Williams, W. M.; Cuvelier, M. E.; Berset, C. L. W. T. Use of a free radical method to evaluate antioxidant activity. *LWT-Food Sci. Technol.* **1995**, *28*, 25-30.

Supplementary Figures

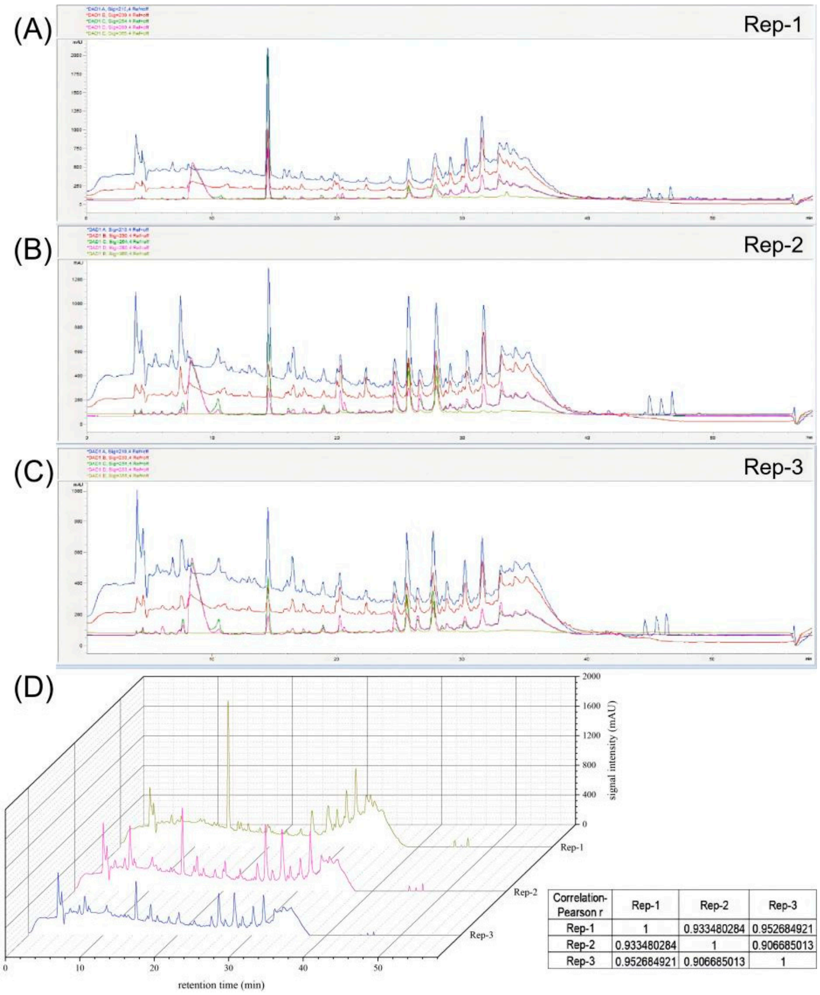

Correlation-Pearson r	Rep-1	Rep-2	Rep-3
Rep-1	1	0.933480284	0.952684921
Rep-2	0.933480284	1	0.906685013
Rep-3	0.952684921	0.906685013	1

Figure S1 HPLC chromatograms for reproducibility assessment. (A–C) HPLC profiles of three parallel replicates Rep-1, Rep-2 and Rep-3. Different colours correspond to distinct UV detection wavelengths: blue at 210 nm, red at 230 nm, green at 254 nm, purple at 280 nm, brown at 365 nm. The x-axis represents retention time (min), and the y-axis represents signal intensity (mAU). (D) Waterfall plots of Rep-1, Rep-2 and Rep-3 acquired at the UV wavelength of 210 nm. Pearson correlation analysis was applied to evaluate the repeatability among samples. The x-axis represents retention time (min), and the y-axis represents signal intensity (mAU).

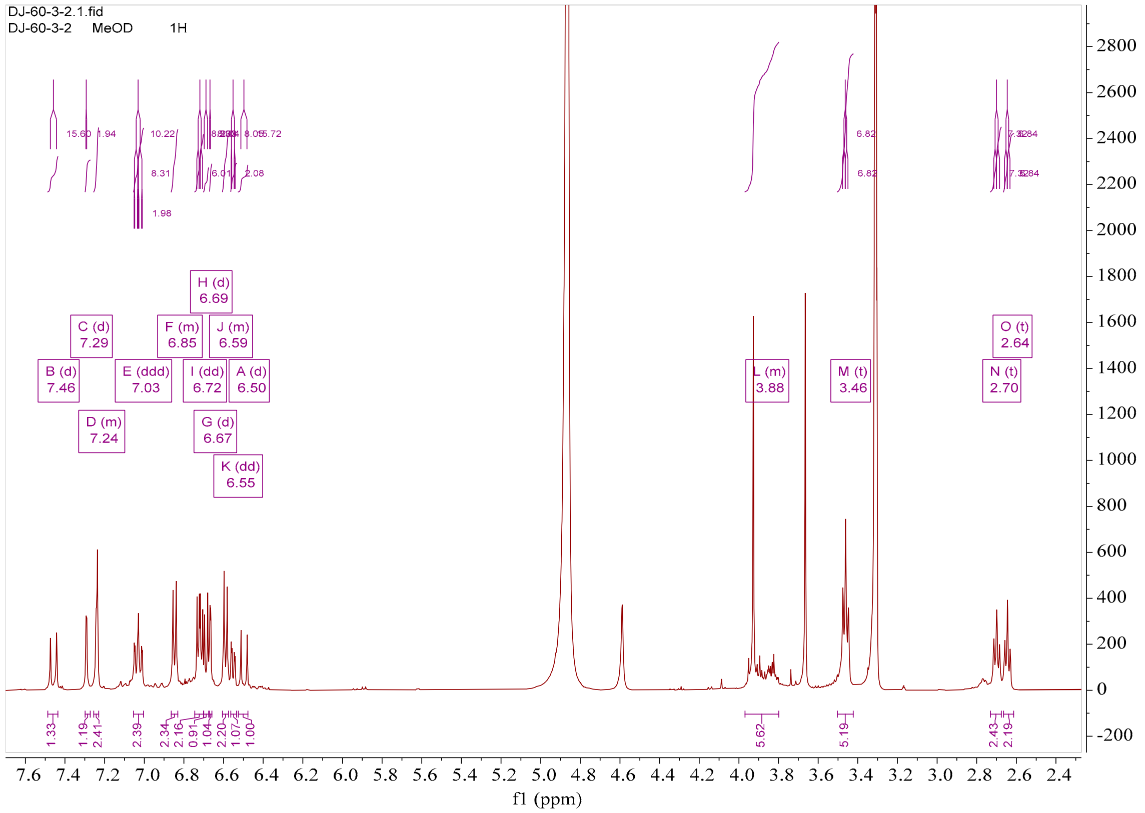

Figure S2 ^{1}H NMR spectrum (500 MHz) of compound 1 in CD_3OD

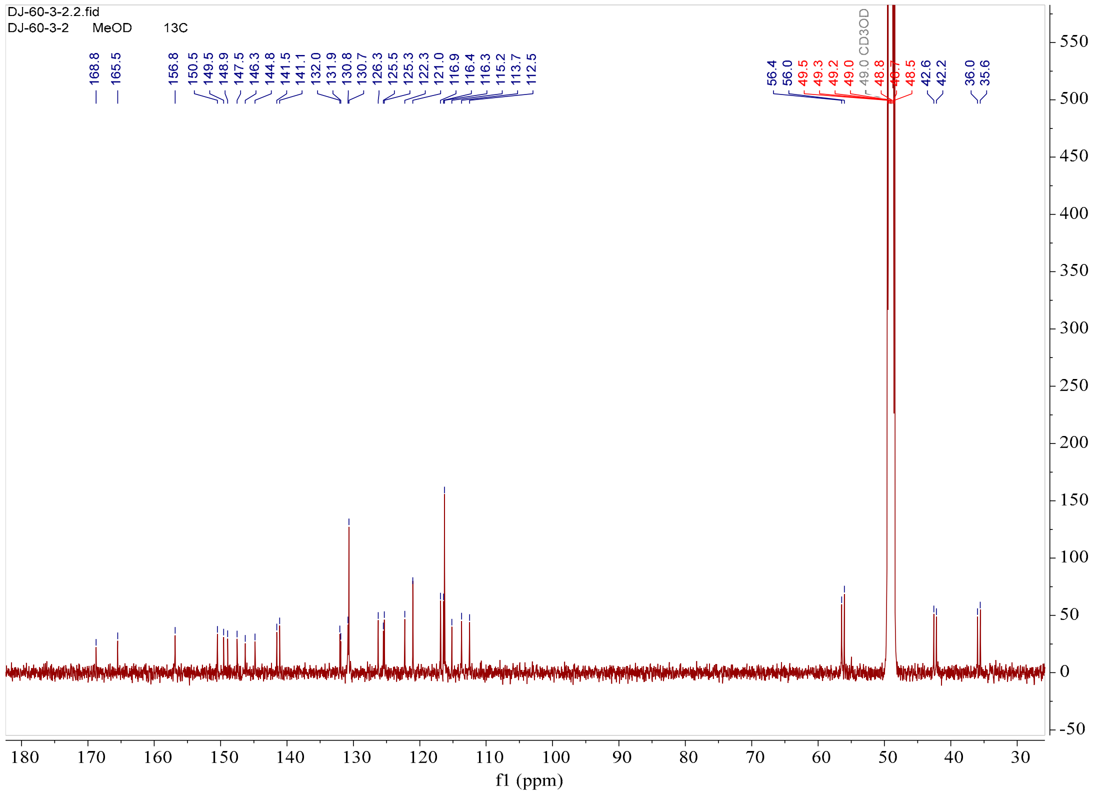

Figure S3 ^{13}C NMR spectrum (125 MHz) of compound 1 in CD_3OD

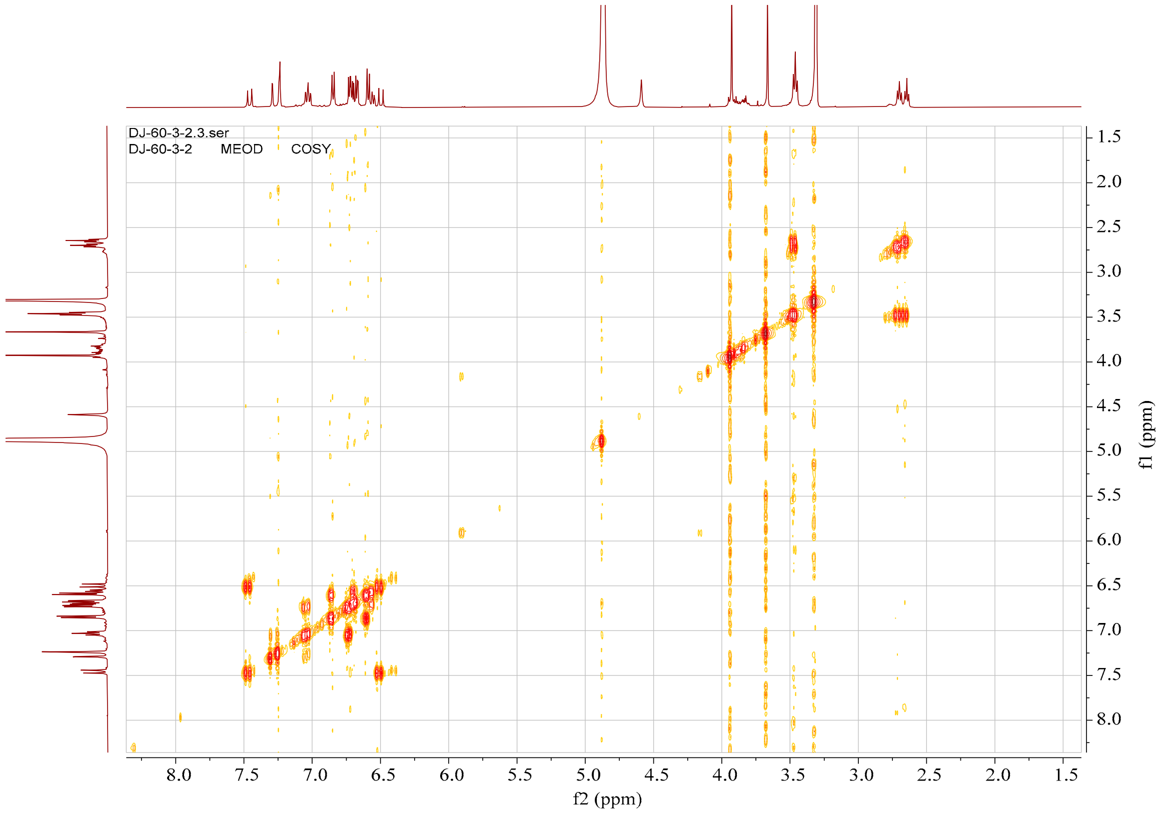

Figure S4 ^{1}H-^{1}H COSY spectrum (500 MHz) of compound 1 in CD_3OD

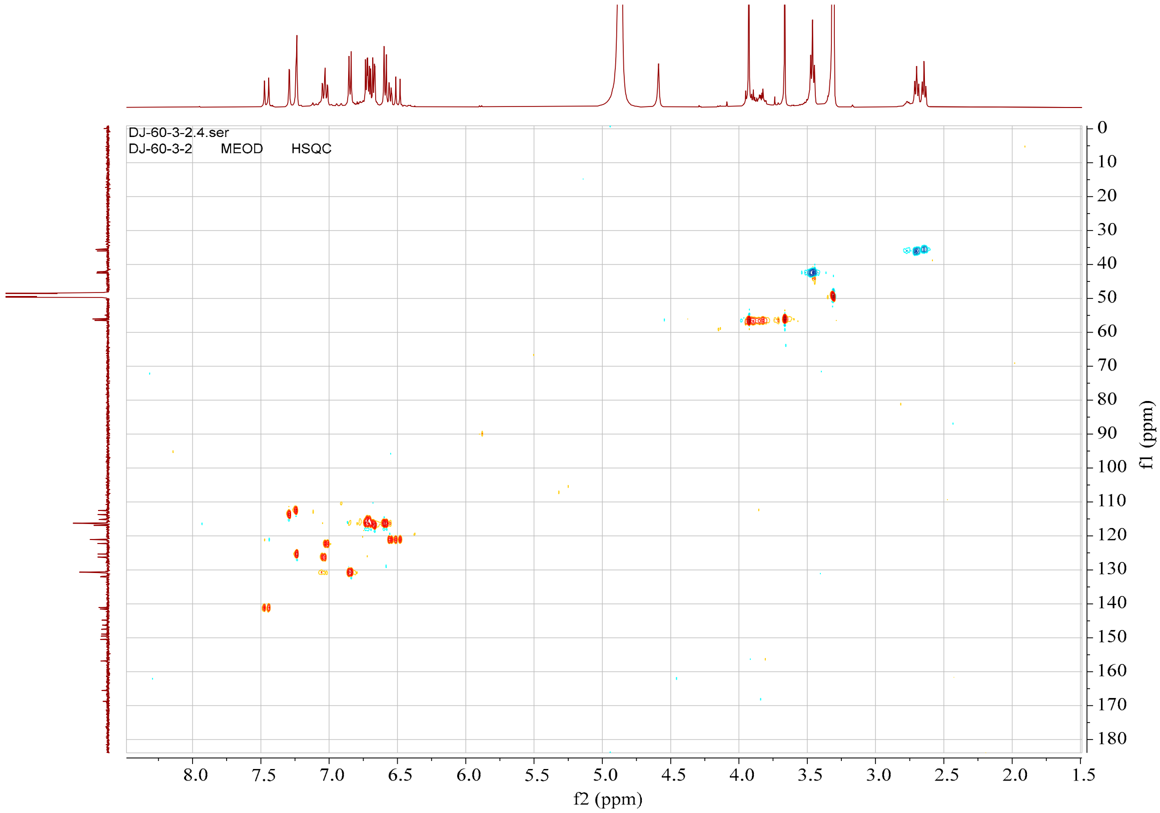

Figure S5 HSQC spectrum (500 MHz) of compound 1 in CD_3OD

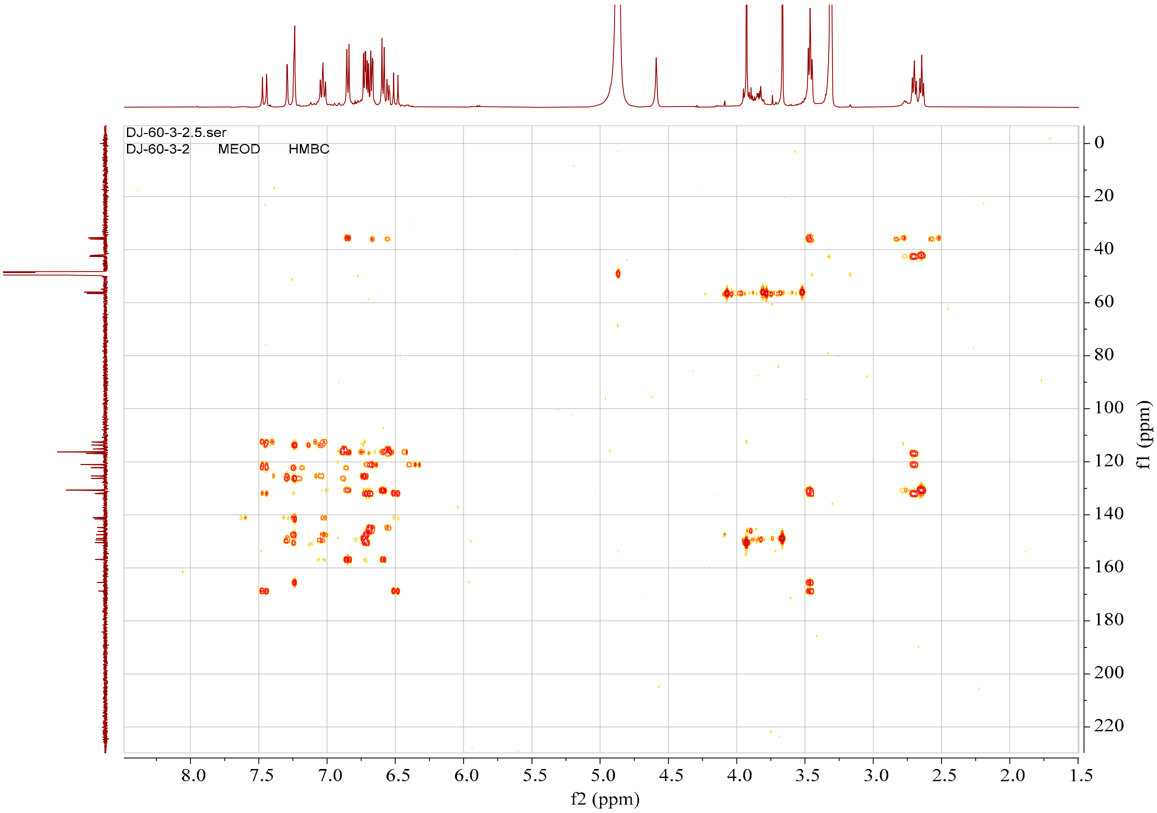

Figure S6 HMBC spectrum (500 MHz) of compound 1 in CD_3OD

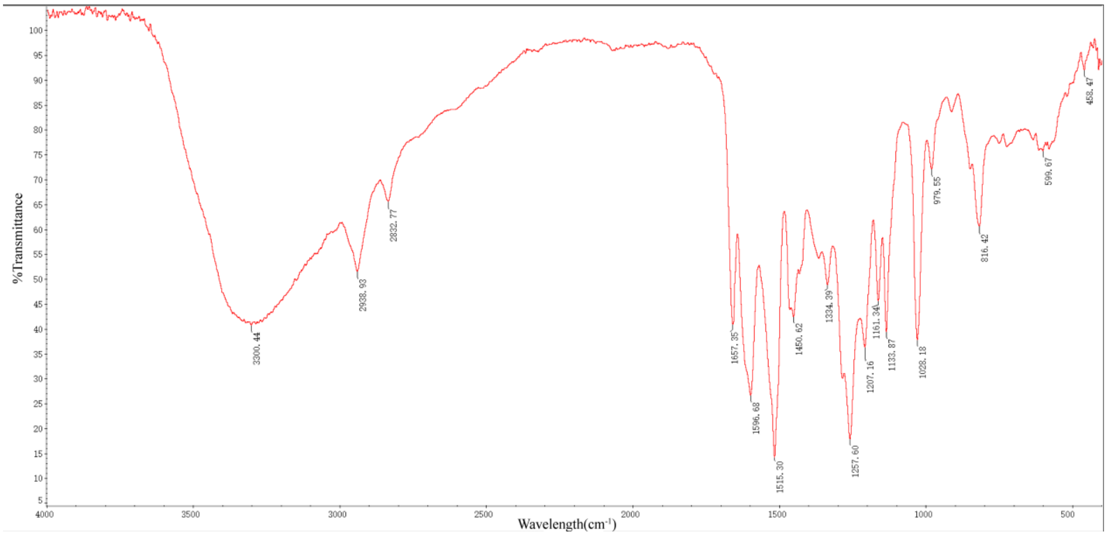

Figure S7 IR spectrum of compound 1

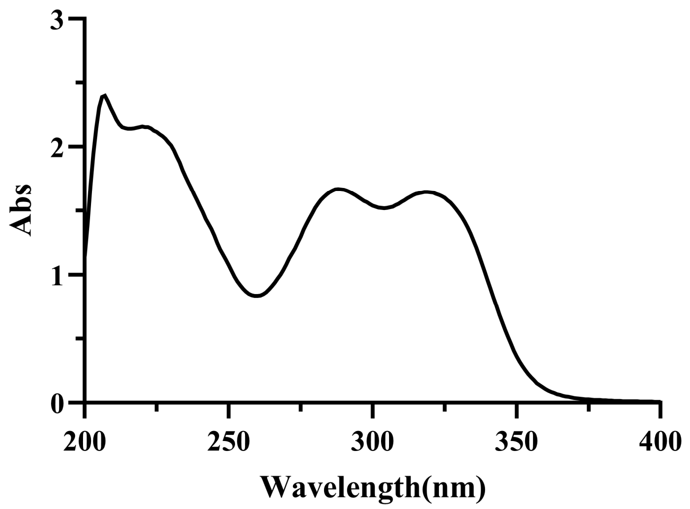

Figure S8 UV spectrum of compound 1 in MeOH

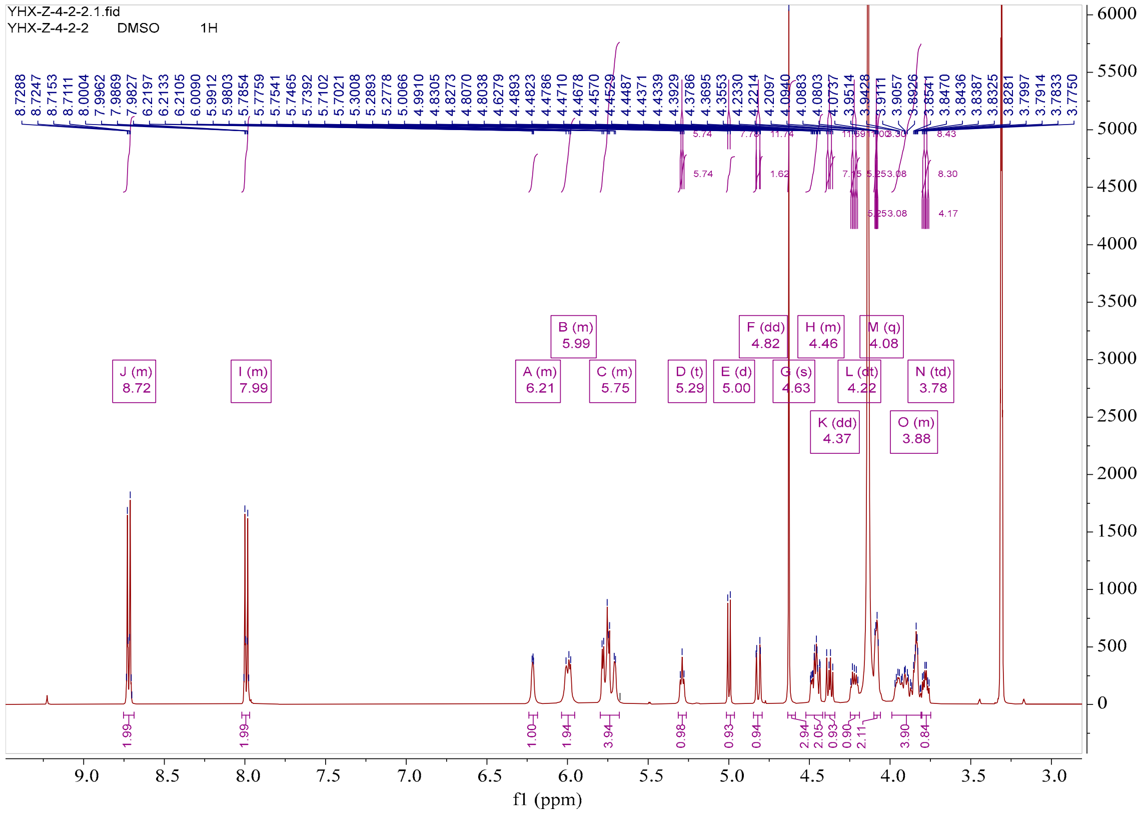

Figure S9 ^{1}H NMR spectrum (500 MHz) of compound 2 in DMSO-d_6

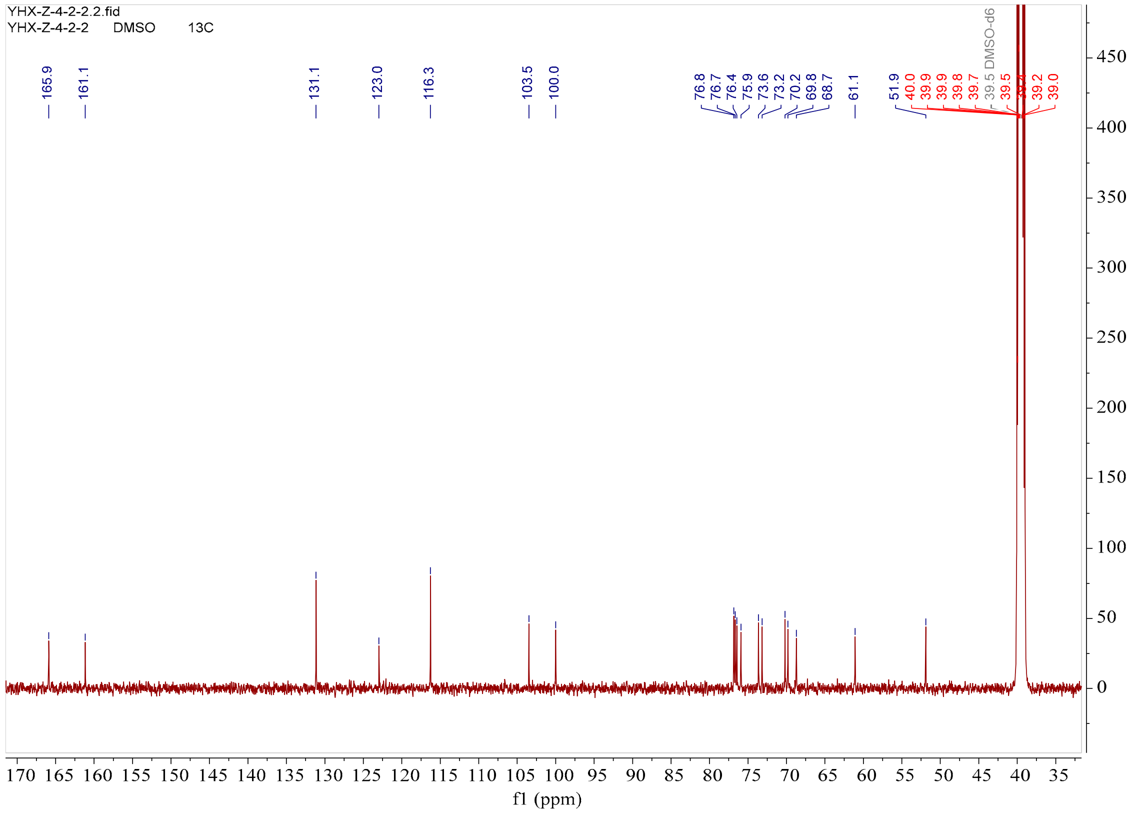

Figure S10 ^{13}C NMR spectrum (125 MHz) of compound 2 in DMSO-*d₆*

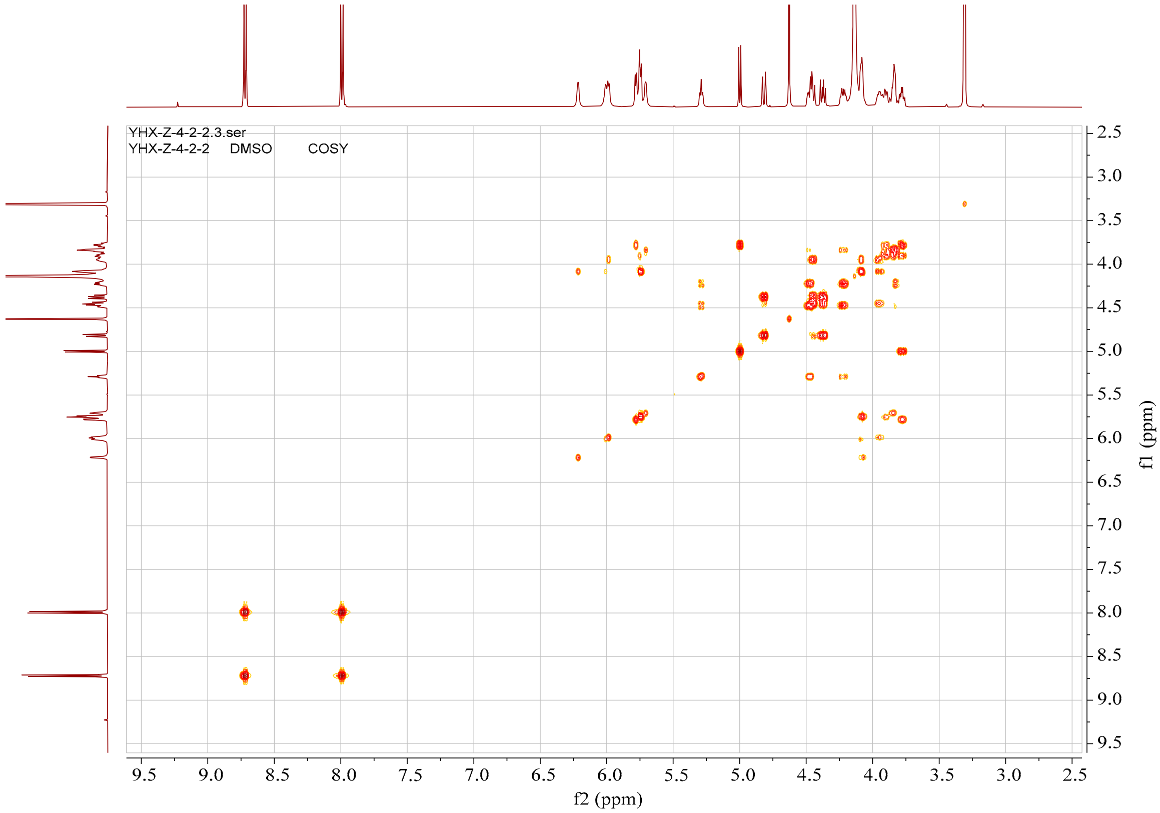

Figure S11 ^{1}H-^{1}H COSY spectrum (500 MHz) of compound 2 in DMSO-d_6

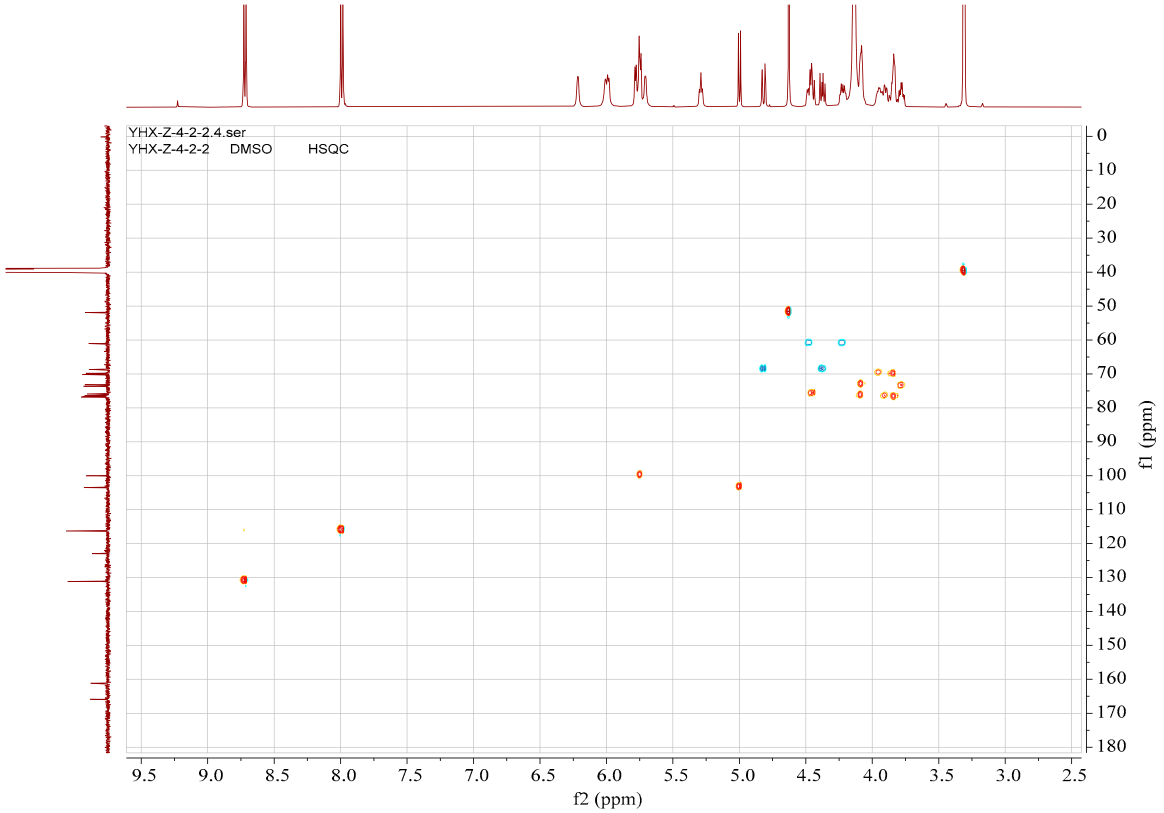

Figure S12 HSQC spectrum (500 MHz) of compound 2 in DMSO-d_6

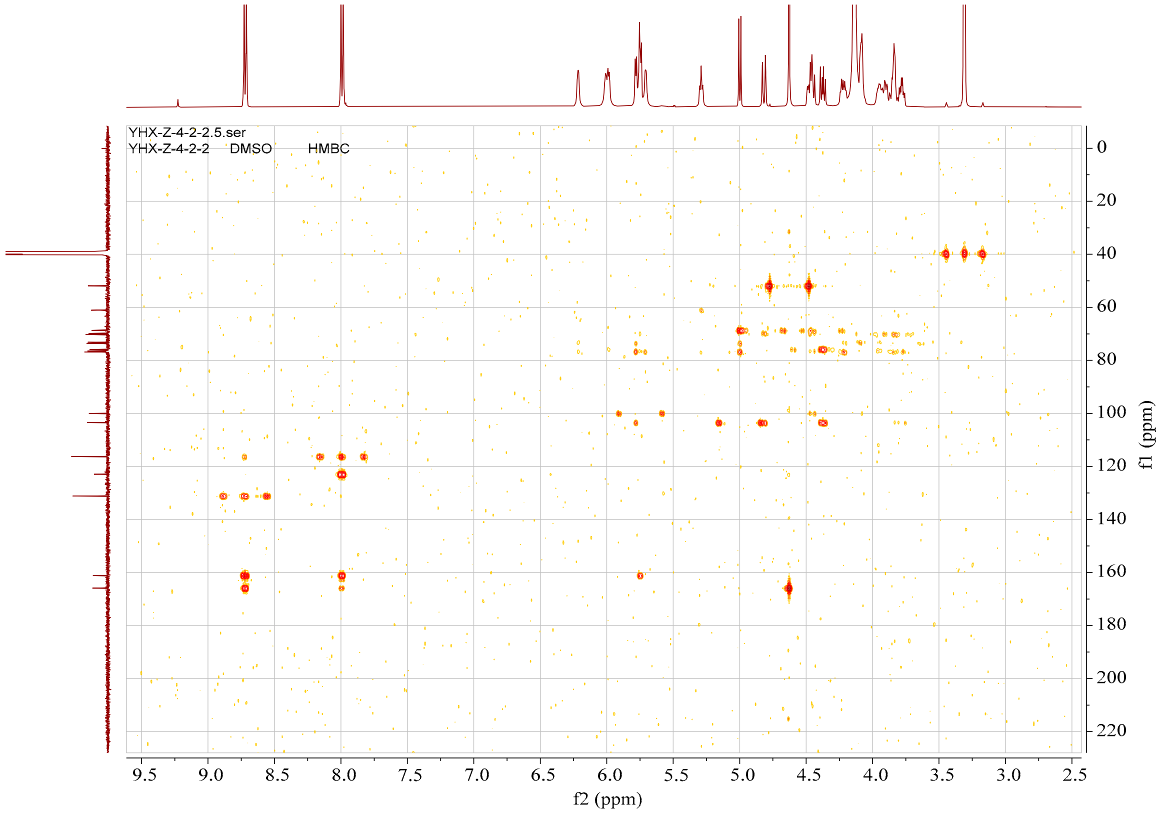

Figure S13 HMBC spectrum (500 MHz) of compound 2 in DMSO-d_6

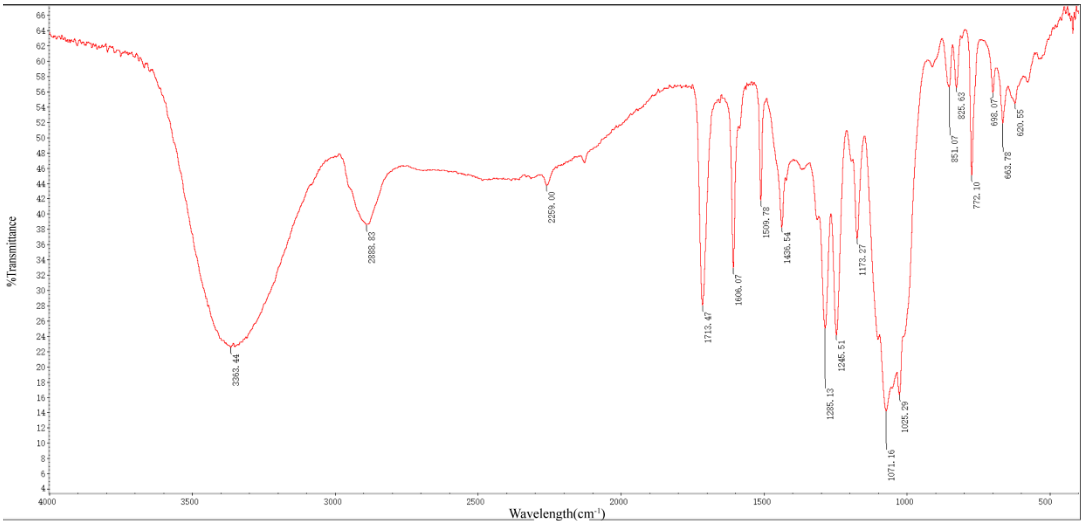

Figure S14 IR spectrum of compound 2

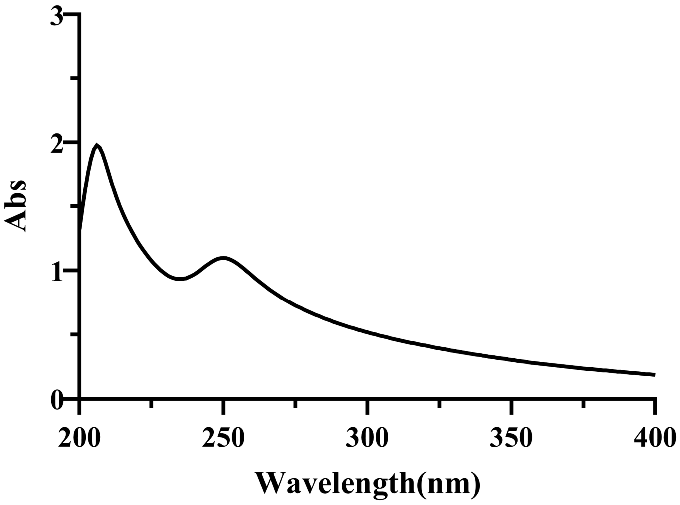

Figure S15 UV spectrum of compound 2 in MeOH

Supplementary Table

Table S1. Antioxidant activities of compounds **3**, **9**, **14** and **18**

**Compund**	**DPPH·IC$_{50}$ (μmol/L)**
ascorbic acid	62.147±9.357
**3**	513.571±122.686
**9**	90.473±12.464
**14**	305.507±15.750
**18**	226.415±56.201
